# Construction of a Rice Glutelin‐Rhamnolipid‐Pectin Nanoparticle Delivery System for Curcumin Delivery: Effect of Pectin Esterification on Characterization, Stability, and Bioaccessibility

**DOI:** 10.1002/fsn3.70823

**Published:** 2025-09-04

**Authors:** Lei Chen, Qiongqiong Wu, Shaokang Li, Shuangli Wang, Weidong Lin, Xiaodan Zang

**Affiliations:** ^1^ College of Public Health Mudanjiang Medical University Heilongjiang China; ^2^ College of Physical Education Mudanjiang Medical University Heilongjiang China

**Keywords:** degree of esterification, pectin, rhamnolipid, rice glutelin

## Abstract

Riceglutelin (RG)‐rhamnolipids (Rha)‐high‐methoxyl pectin (HMP)/medium‐methoxy pectin (MMP)/low‐methoxy pectin (LMP) were used to attract self‐assembled nanoparticles by electrostatic attraction, and the effects of pectin esterification degree and concentration on the characteristics of nanoparticles and the bioavailability of curcumin were evaluated. The minimum particle size and the highest encapsulation efficiency of the nanoparticles were at mass ratios of RG:HMP = 2:1, RG:MMP = 4:1, and RG:LMP = 2:1. The results of Fourier transform infrared spectroscopy and circular dichroism spectroscopy show that hydrogen bonding, hydrophobic interaction, and electrostatic attraction are the main driving forces for the formation of complexes. The X‐ray diffraction results verified that the curcumin in the complex was in an amorphous state. In addition, curcumin embedded with nanoparticles has good photothermal and storage stability. The results of in vitro simulated digestion showed that nanoparticles constructed from LMP had high bioaccessibility. The results of this study are expected to provide new ideas for improving the bioaccessibility of curcumin.

## Graphical abstract

Construction of nanoparticles using alkali heat treatment of rice glutelin and further successful construction of nanoparticles encapsulating curcumin using electrostatic mutual attraction. The FTIR, CD, and XRD results revealed that electrostatic, hydrogen bonding, and hydrophobic interactions were involved in the construction of the nanoparticles. The constructed rice glutelin‐based nanoparticle delivery system has good light, heat stability, and storage stability. The constructed rice glutelin‐rhamnolipid‐low‐methoxyl pectin nanoparticles had better curcumin bioaccessibility.

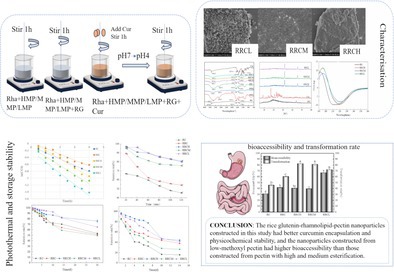

## 1 Introduction

Curcumin (Cur) is a promising therapeutic agent that has a broad spectrum of pharmacological effects; its multifaceted pharmacological properties, including anticarcinogenic, hypoglycaemic, anti‐inflammatory, and antioxidant activities, have led to its extensive evaluation in numerous clinical trials. For example, Rivera‐Mancía discussed the hypoglycaemic effects of Cur (or its analogs) and its effectiveness as an adjuvant to improve diabetes and related complications (Rivera‐Mancía et al. 2018). Wang investigated the cardioprotective effects of Cur in diabetic cardiomyopathy (Wang, Chang, et al. 2024). Cur's potential to lower depression and atherosclerosis risk in obese individuals with type 2 diabetes mellitus was investigated by Yaikwawong in a randomized controlled trial (Yaikwawong et al. 2024a, 2024b). Muhammadi investigated the therapeutic effects of Cur in the management of atopic dermatitis (Mohammadi et al. 2024). Moreover, Zhao discussed the primary mechanism by which Cur reduces inflammatory cytokine production by blocking signaling pathways to alleviate inflammatory symptoms (Zhao 2024). All of these studies reveal the potential of Cur to ameliorate or alleviate many of the diseases currently afflicting humanity. Cur is also nontoxic, readily available, and very affordable. However, Cur has low bioavailability due to its poor water solubility, photosensitivity, and physicochemical stability, significantly limiting its application in biomedicine and food health benefits. Nanotechnology has become an extensively studied tool commonly used to improve the application of hydrophobic active ingredients. Recent studies have also demonstrated that nanoparticle‐based delivery systems can effectively improve the oral bioavailability of these ingredients.

Rice protein (RP) is considered a high‐quality source of plant protein because of its hypoallergenic properties, high digestibility, hypocholesterolaemic properties, and good biological value (Dai et al. 2017). RG constitutes 80% of rice protein and is the main component of RPs. Despite the many advantages of RGs, they contain many hydrophobic amino acids, leading to poor water solubility, and tend to aggregate in large quantities, limiting their wide application (Li, Wang, et al. 2024). Many methods (e.g., physical treatments, chemical modifications, and enzymatic treatments) have been used to modify the structure of RGs to improve the solubility and functionality of proteins. Surfactants have been demonstrated to increase the stability and capacity of protein‐based nanocomposites to transport hydrophobic bioactive compounds while causing protein molecules to group together to form more compact structures (Gao et al. 2024). The anionic glycolipid biosurfactant rhamnolipid (Rha), which is primarily generated from *Pseudomonas aeruginosa* , has been employed extensively to create very stable and dispersible inorganic nanoparticles. Specifically, it was found that rhamnolipids as a constituent material for nanoparticles have a number of advantages; for example, they have been widely used in the preparation of nanoparticles with high stability and dispersibility, and they also have some antibacterial properties, which can increase the functionality of the nanoparticles (Ali et al. 2024). Furthermore, it can induce protein molecules to aggregate into clusters, thus forming a more compact structure (Guo et al. 2021). Therefore, the addition of rhamnolipids can improve the ability and stability of protein‐based composite nanomaterials to carry hydrophobic actives. It has been previously shown that the surfactant rhamnolipid enhances the encapsulation rate and stability of zein, pea protein, and soy protein nanoparticles (Dai, Li, et al. 2018; Gao et al. 2024; Guo et al. 2020). By hydrophobic, electrostatic, and hydrogen bonding interactions, polysaccharides are typically adsorbed on the surface of partially unfolded and self‐aggregated protein particles, improving the stability of the proteins and making them appropriate for use as materials for nanodelivery systems. Polysaccharides are also known for their low toxicity, good biocompatibility, and biodegradability (Su et al. 2021). Pectin is an anionic polysaccharide widely used in food and pharmaceutical applications, which is able to protect against pepsin and gastric acid environments and has a positive effect on reducing cancer (Cheng et al. 2024). Pectin‐containing covered liposomes or nanoparticles have a certain stability that enhances the bioavailability of nutrients (Chang et al. 2017). Studies have shown that pectins with different degrees of esterification have different functions, such as viscosity and molecular weight, while the degree of esterification of pectin significantly affects the physicochemical properties of the constructed complexes (Guo et al. 2020). Numerous studies have shown that protein–polysaccharide–surfactant nanoparticle delivery of hydrophobic active ingredients via noncovalent interactions is a promising and effective method for improving protein function and active ingredient bioavailability. For example, Gao prepared a Rha/SSPS/SPI nanodelivery system with a small particle size, high drug loading, and release characteristics (Gao et al. 2024). Guo prepared ternary complexes of rhamnolipids with pea isolate proteins and high‐methoxyl pectin for the coencapsulation of Cur and resveratrol, which resulted in relatively small particle sizes and high loadings (Guo et al. 2021). Compared with the binary complex, the ternary nanoparticle complex of zein–propylene glycol alginate–rhamnolipid constructed by Dai can effectively encapsulate and deliver Cur, and the presence of polysaccharides and surfactants not only improves the encapsulation rate of Cur but also significantly improves the photostability and bioaccessibility of Cur (Dai, Wei, et al. 2018). Numerous studies have demonstrated that the presence of polysaccharides and surfactants increases the encapsulation rate of active ingredients and significantly improves their physicochemical stability and bioaccessibility. There are few studies on glutelin‐based nanodelivery systems, especially on glutelin‐rhamnolipid‐pectin nanodelivery systems for embedding hydrophobic active components.

For these reasons, to increase the bioavailability of Cur, a delivery system constructed from rice glutelin‐rhamnolipid‐pectin with different degrees of esterification was prepared in this study. The effects of the degree of pectin esterification on the structure of the nanodelivery system were investigated via Fourier transform infrared (FTIR) spectroscopy, circular dichroism (CD) spectroscopy, and X‐ray diffraction (XRD) spectroscopy. Scanning electron microscopy (SEM) was also used to characterize the external appearance. Light, heat, and storage stability were also evaluated. Finally, its ability to deliver Cur was studied to assess its bioavailability. The study of the application of Cur in functional foods is expected to provide new references for improving the bioavailability and functional properties of Cur and RG.

## 2 Materials and Methods

### 2.1 Materials

RG was extracted from our laboratory, and the protein content was 83.3% (according to the Kjeldahl method). Rhamnolipid (Rha) with 43.34% purity was obtained from Aladdin Biological Technology (Shanghai, China). High‐methoxy pectin (HMP) (DE value 70%–77%), medium‐methoxy pectin (MMP) (DE value 58%–62%) and low‐methoxy pectin (LMP) (DE value 36%–40%) were purchased from Yantai Andre Pectin Co. Ltd. (Shandong, China). Curcumin (Cur) (98%) was purchased from Macklin (Shanghai, China). Other chemicals were of analytical grade.

### 2.2 Construction of a Complex of RG‐Rha‐HMP/MMP/LMP of Encapsulated Cur

#### 2.2.1 Solution Preparation

The RG solution (2%, w/w) was obtained by alkaline–thermal cotreatment according to the description of Guo et al. (2021) with appropriate modifications. Briefly, RG (2 g) was dispersed in deionized water (0.1 kg) and stirred for 30 min. Thereafter, the pH of the dispersion was adjusted to 12 with NaOH, and it was then stirred for another 30 min. Then, the dispersion was heated at 65°C for 30 min to dissolve completely. Subsequently, the aqueous RG solution was rapidly cooled to 23°C, and the pH was reduced to 7.4. Four percent solutions of pectin with different degrees of esterification were prepared by dissolving HMP (2 g), MMP (2 g), and LMP (2 g) in 0.05 kg of deionized water under stirring. Rha (1 mg/mL, w/v) dissolved in deionized water was also allowed to dissolve completely. The pH of each aqueous solution was subsequently adjusted to 7.4, and all the aqueous pectin solutions were diluted to different concentrations so that RG:HMP/MMP/LMP = 10:1, 4:1, 2:1, 1:1, or 1:2. A Cur solution (5 mg/mL) was obtained by dissolving Cur in 1 mL of anhydrous ethanol.

#### 2.2.2 Preparation of Nanoparticles of Encapsulated Cur

The ternary complexes of encapsulated Cur were prepared as follows: 20 mL of Rha solution (1 mg/mL, pH 7.4) was added to 10 mL of HMP, MMP, and LMP solutions of different concentrations (pH 7.4). After stirring for 60 min, 10 mL of RG solution (1 mg/mL, pH 7.4) was added to the above solutions, and stirring was continued for 60 min. Then, 1 mL of Cur alcohol solution was added to each of the above mixed solutions, followed by stirring again for 60 min. The final pH of all the solutions was adjusted to 4.0 to form the Cur‐loaded ternary complex. Finally, the suspension was centrifuged (3000 *g*, 15 min) via a centrifuge (Sigma 3 k15, Osterode am Harz, Germany). RG‐Rha‐Cur‐HMP/MMP/LMP were used to construct nanoparticles denoted RRCH/RRCM/RRCL, respectively.

#### 2.2.3 Preparation of Control Samples

Previously, RG‐Cur (RC) and RG‐Rha‐Cur (RRC) nanoparticles were prepared in our laboratory via a pH‐driven method as a control, and their photothermal stability and bioaccessibility were also studied in a controlled manner.

RG powder was dissolved in distilled water via a magnetic stirrer to form a 10 mg/mL dispersion. The pH of the mixture was increased to 12, and the mixture was stirred for 2 h. A proportion of Cur powder was subsequently added to the RG solution at pH 12, with a final mass ratio of RG to Cur of 40:1. The resulting mixture was mixed with an equal volume of Rha solution (5:1 mass ratio of RG‐Rha) and stirred for 30 min. The pH was subsequently adjusted to 7, and the mixture was stirred for another 30 min to form composite nanoparticles containing nutraceuticals. The resulting nanoparticle dispersion was centrifuged (3000 *g*, 15 min) to eliminate larger particles and unbound Cur.

### 2.3 Characterization of Complexes Constructed From RG‐Rha‐HMP/MMP/LMP of Encapsulated Cur

#### 2.3.1 Particle Size, Zeta (ζ)‐Potential and Polydispersity Index (PDI) Measurement

The particle size, ζ‐potential, and PDI of the above nanoparticles with different mass ratios were examined via a Zetasizer Nano‐ZS90 (Malvern Instruments Ltd., Worcestershire, UK).

#### 2.3.2 Fluorescence Spectroscopy (FS)

Briefly, the FSs of the mixture samples were recorded at an excitation wavelength of 280 nm and excitation and emission slit widths of 2.5 nm. An RF‐6000 fluorescence spectrophotometer (Hitachi High‐Tech Corporation, Tokyo, Japan) was used to characterize changes in the local environment of the tyrosine and tryptophan residues in RG.

#### 2.3.3 Ultraviolet (UV) Spectra

Ultraviolet (UV) spectra were obtained with a UV‐1000 spectrophotometer (Beijing Lebertec Instruments, Beijing, China) with a scanning range of 220–5000 nm and a scanning rate of 200 nm/min.

The selection of nanoparticles with the optimal mass ratio for further studies was based on the particle size potential, fluorescence spectroscopy, and UV spectroscopy data.

### 2.4 Encapsulation Properties of the Nanoparticles

The Cur content was calculated via Guo's procedure with simple modifications (Guo et al. 2021). Using a spectrophotometer (Beijing Lebertec Instruments, Beijing, China), the Cur concentration in the upper organic phase was determined at 426 nm. In brief, 0.4 mL of sample was mixed with 3 mL of extraction solvent (10:1 v/v ethyl acetate: absolute ethyl alcohol), and the mixture was combined until distinct layers formed. The following formulas were used to determine the encapsulation efficiency (EE) and loading capacity (LC):

$$\mathrm{EE}\left(\%\right)=\text{encapsulated}\;\mathrm{Cur}/\text{total}\;\mathrm{Cur}\;\text{input}\times 100\%$$

$$\mathrm{LC}\left(\%\right)=\text{encapsulated}\;\mathrm{Cur}/\text{total}\;\mathrm{RG}\;\text{and surfactant and pectin input}\times 100\%$$

### 2.5 Driving Forces for Nanoparticle Formation

Equal volumes of composite nanoparticle suspensions were mixed with solutions of sodium dodecyl sulfate (SDS), urea, or dithiothreitol (DTT) at different concentrations, and the mixtures were allowed to stand at room temperature for 6 hours. The absorbance at 600 nm was measured via a UV spectrophotometer, and the concentration gradients of1 to 55 represented the following: SDS (0.05%, 0.1%, 0.15%, 0.2%, and 0.25%), urea (1, 2, 3, 4, and 5 M) and DTT (10, 20, 30, 40, and 50 mM). The turbidity of the mixture after standing was measured via the suspension turbidity measurement method described in Section 2.4. The relative change in turbidity indicates the degradation of the composite nanoparticles.

### 2.6 Fourier Transform Infrared Spectroscopy (FTIR)

A total of 1.0 mg of sample was taken separately, mixed, and ground with 80 mg of KBr. After the tablets were pressed, the infrared spectra of different substances were measured in the wavelength range of 400–4000 cm^−1^ via a Fourier transform infrared spectrometer (Thermo Scientific Nicolet is5 FTIR, Massachusetts, USA), which was calibrated for background signal correction.

### 2.7 Circular Dichroism (CD)

The far‐ultraviolet CD spectra were recorded via a CD spectrophotometer (Chirascan, Leatherhead, UK) according to the method described by Arroyo‐Maya et al. (2016). All the spectra were scanned in the far‐ultraviolet range between 190 and 250 nm at room temperature (25°C ± 1°C) with continuous nitrogen purging during data acquisition. The protein concentration was set to 0.2 mg/mL, and the path length was 0.1 cm. The ellipticity was recorded at a speed of 100 nm/min, with a resolution of 0.2 nm, 20 accumulations, and a bandwidth of 2.0 nm. Changes in the contents of α‐helices, β‐folds, β‐turns, and random curls of RGs were calculated via the CONTIN method.

### 2.8 X‐Ray Diffraction (XRD)

The XRD patterns of the nanoparticles were examined via an X‐ray diffractometer (D8 ADVANCE A25; Bruker, Germany). They were collected at a 30 kV voltage and 10 mA current using 40 kV Cu‐Kα radiation and scanned at 2°/min. The scanning range was 5°–600°, and the diffraction angle was set to 2θ.

### 2.9 Scanning Electron Microscopy (SEM)

The lyophilized samples were fixed and then gold‐plated on their surfaces by ion sputtering (to prevent the samples from charging under the electron beam), and then placed under a scanning electron microscope (SU8010, Hitachi High‐Tech Corporation, Tokyo, Japan) to observe their morphology at a voltage of 5.0 kV.

### 2.10 Photothermal and Storage Stability Studies

#### 2.10.1 Photodegradation

The Cur‐embedded nanoparticles were dispensed in transparent glass vials and placed under natural light for 4 h at room temperature. The amount of Cur in the nanoparticles was determined hourly. The degradation rate constant (*k*) and half‐life (*t*_1/2_) were calculated via the first‐order kinetic equation as follows:

$$\ln\left(C/C_{0}\right)=-\mathrm{kt}$$

$$t_{1/2}=\ln2/k$$

#### 2.10.2 Thermal Stabilization

The nanoparticles loaded with Cur were incubated at 80°C for 2 h. The contents of Cur and the nanoparticles were measured every 20 min, and the retention of Cur was calculated via the following equation:

$$\text{Retention rate}\left(\%\right)=\frac{\text{remaining curcumin in nanopartics}}{\text{initial curcumin in nanoparticles}}\times 100\%$$

#### 2.10.3 Storage Stability

Storage stability was determined by monitoring the change in retention of Cur when the nanoparticles were stored at room temperature for 14 days and at 4°C for 30 days.

### 2.11 Determination of Cur Bioaccessibility and Transformation Rate

During mock digestion, 20 mL of the sample was first added to 20 mL of SGF, followed by stirring, preheating to 37°C, and digestion for 1 h, after which the pH was adjusted to 7. The gastric digestion products described above were then mixed with an equal volume of the mock intestinal mixture, and digestion was terminated by an ice bath after 2 h of digestion at 37°C. The digested sample was then incubated at 4°C and centrifuged (10,000 *g*, 15 min) to obtain the intermediate clear micelle phase. One milliliter of intermediate clear micelle phase was mixed with 3 mL of extractant. The absorbance of the solution was measured at 426 nm. The Cur concentration was analyzed via a standard curve.

Bioaccessability%=CmicelleCdigest×100%

Transformation rate%=CdigestCinitial×100%

Here, *C*_micelle_ represents the amount of Cur in the micelles, *C*_digest_ represents the amount of Cur in the digestive solution, and *C*_initial_ represents the amount of Cur in the initial nanoparticles.

### 2.12 Statistical Analysis

The results were analyzed via the mean and standard deviation, and each experiment was conducted three times. The significant differences were examined via one‐way analysis of variance (ANOVA) and the Duncan model. *p* < 0.05 was used to indicate a significant difference.

## 3 Results and Discussion

### 3.1 Characterization of RG‐Rha‐HMP/MMP/LMP

#### 3.1.1 Particle Size, PDI and ζ‐Potential

**FIGURE 1:**
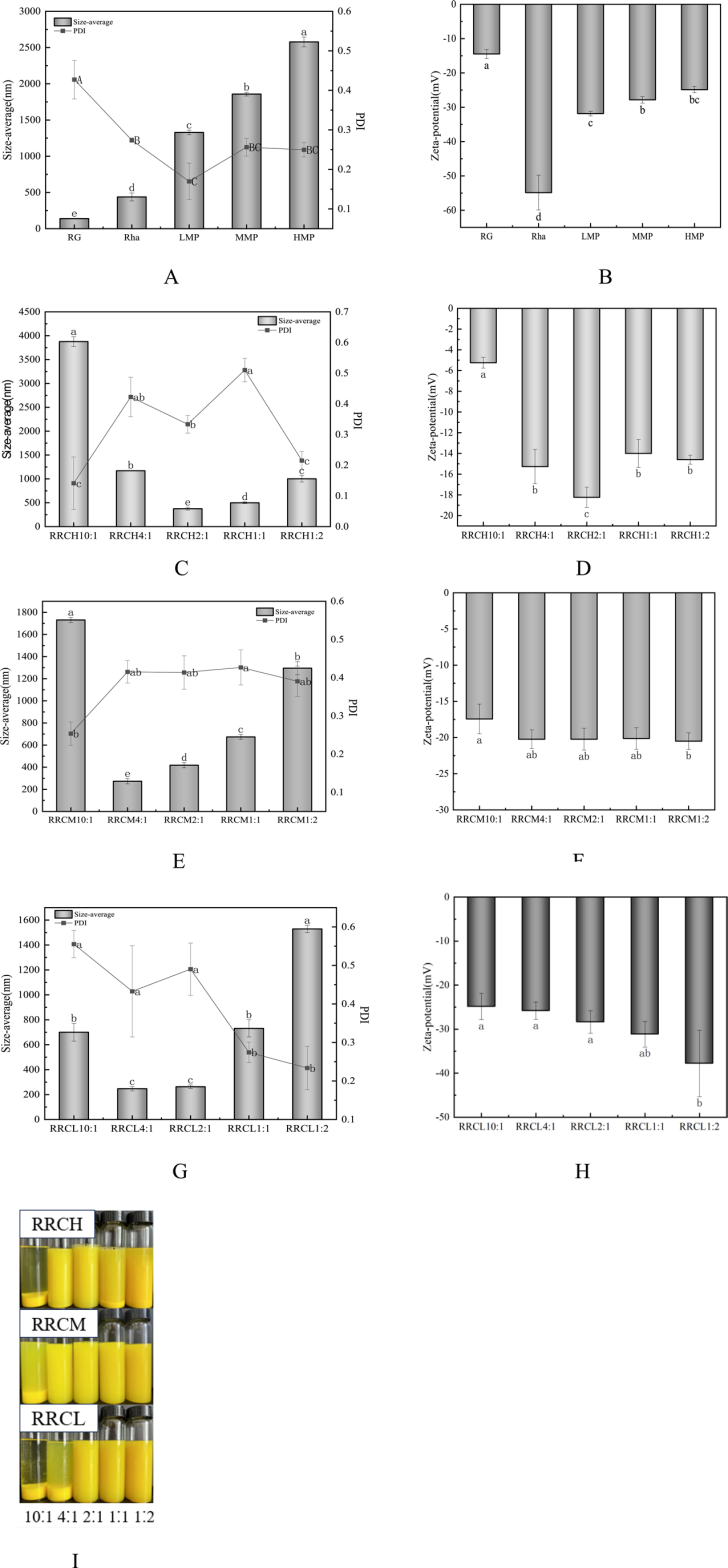
Particle size, ζ‐potential, and PDI for RG, Rha, LMP, MMP, and HMP (A, B). Effect of the mass ratio of rice glutelin to HMP (C, D)/MMP (E, F)/LMP (G, H) on the mean particle size, PDI, zeta potential, and visualization (I) of composite nanoparticle dispersions. The data are displayed as the means ± SDs (*n* = 3), with different letters denoting statistically significant differences (*p* < 0.05).

The particle size and ζ‐potential of the individual substances were shown in Figure 1A,B. As the degree of esterification increases, more free carboxyl groups with anions in arein were replaced by methylation, resulting in a gradual increase in particle size and a decrease in potential (Schmidt et al. 2017). As can be seen in Figure 1C,E,G, the particle size of the nanoparticles tended to decrease and then increase with increasing pectin concentration at the same level of pectin esterification. The visualization shown in Figure I also indicated that high concentrations oandboth glutelin or HMP/MMP/LMP lead to the creation of precipitates and larger aggregates, resulting in an increase in particle size. Particle size results reflected two competing mechanisms: (1) At low pectin concentrations, insufficient charge density (particularly for high‐esterification pectin) limits complex formation. (2) Excess pectin forms a surface layer that reduces interparticle repulsion and promotes protein self‐association through enhanced hydrophobicity (Gu et al. 2024; Zhan et al. 2018). Thus, RG with HMP/MMP/LMP at suitable mass ratio conditions would yield compounds with relatively small particle sizes. Nanoparticles with smaller particle sizes were found at RG:HMP = 2:1, RG:MMP = 4:1, and RG:LMP = 2:1. Particle sizes of (374.4 ± 31.1), (273.4 ± 23.5), and (262.7 ± 13.9) nm, respectively. These results demonstrated that pectin with low esterification and a high charge density produced nanoparticles with smaller particle sizes, which was similar to the findings of Cheng (Cheng et al. 2024). Combined with the potentiometric results, the potentials of RRCH2:1/RRCM4:1/RRCL2:1 are intermediate between those of individual proteins, rhamnolipids, and pectins, and this electrically neutralized phenomenon suggests that electrostatic attraction is involved in the formation of nanoparticles (Guo et al. 2020). It has also been shown that higher charge density produces stronger attraction leading to a more compact structure and thus a smaller particle size of RRCL2:1 in the RRCH2:1/RRCM4:1/RRCL2:1 complex. The above results showed that electrostatic interaction was responsible for the formation of the nanocomplexes, which was among the main driving forces. The PDI of the nanoparticles showed the distribution of the particles in solution, and the PDI of the nanocomplexes with smaller particle sizes was also relatively small, suggesting that the distribution of the particles in solution at this point in time was also relatively homogeneous.

The potential of the nanoparticles was shown in Figure 1. As can be seen in Figure 1D,F,H, the average potential of the nanocomplexes tended to decrease as the degree of pectin esterification increases, which was in agreement with the results of Guo's study (Guo et al. 2020). The ζ‐potential profiles exhibited distinct esterification‐dependent behaviors; specifically, HMP demonstrated a nonmonotonic trend (initial increase followed by a decrease Figure 1D), reaching a maximum negative potential at the optimal HMP:RG ratios (2:1 w/w), where enhanced electrostatic repulsion minimized particle agglomeration. In contrast, MMP/LMP showed concentration‐dependent monotonic potential increases (Figure 1F,H), attributed to their greater free carboxyl group (‐COOH) availability and consequently higher charge density. When the pectin concentration continues to increase, the excess pectin molecules covered the surface of the aggregates with a shielding function (Šurlan et al. 2023), leading to a decrease in the absolute value of the ζ‐potential. The final potentials were all approximately equal to the potential of the pectin itself, indicating that, after a certain pectin concentration was reached, all shells in the outer layer of the nanocomplexes The were formed. RRCH potential trend was different from RRCM/RRCL (Figure 1D,F,H) can be explained by two reasons: first, HMP has fewer free carboxyl groups, resulting in a lower potential; and second, HMP bridges helical chains at the interface through hydrogen bonding and hydrophobic interactions (Schmidt et al. 2017); this leaded to a greater shielding effect, resulting in a decrease in the absolute value of the negative ζ‐potential, which also explained the gradual decrease in the absolute value of the potential of the nanoparticles as the degree of pectin esterification increases at the same pectin concentrationresultstential resultes again indicated that the intermolecular electrostatic interaction force was one of the main drivers of nanoparticle composition.

#### 3.1.2 FS

**FIGURE 2:**
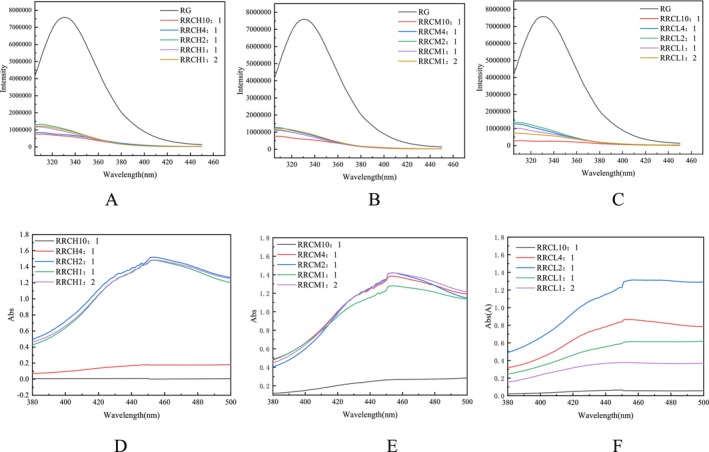
Fluorescence spectra of different mass ratios of rice glutelin with HMP (A)/MMP (B)/LMP (C) contents. UV spectra of different mass ratios of rice glutelin with HMP (D)/MMP (E)/LMP (F) contents.

The fluorescence spectra of the nanoparticles constructed with different concentrations and different degrees of esterification of pectin were shown in Figure 2A–C. The figure showed that under the excitation wave condition of 290 nm, the maximum emission peak of RG, which was the tryptophan residue in RG, appeared at approximately 335 nm (Li, Wang, et al. 2024). When the nanocomplexes embedded with Cur were successfully constructed, the fluorescence emission peaks decreased significantly, and the peaks appeared blueshifted, indicating that more hydrophobic groups were exposed. These phenomena can be explained as follows: (I) Cur binds to the hydrophobic sites of glutelin, leading to fluorescence quenching. (II) The presence of polysaccharides leads to a decrease in the fluorescence intensity of nanoparticles. (III) Rhas and Cur bind to glutelin through hydrophobic interactions, causing the structure of the protein to be more stretched, thus exposing more hydrophobic sites. (IV) Introduction of Rha hydrophobic groups increases the hydrophobicity of the nanoparticles. At a glutelin to pectin ratio of 10–11, all the nanoparticles presented low peaks, indicating that the solution was almost nonfluorescent (Dai et al. 2019), a phenomenon that could be attributed to the production of macroaggregates resulting in a low concentration of proteins in the solution, as shown in Figure 1I. At other ratios, the peaks tended to decrease as the pectin content increased, which may be due to the gradual stretching of the protein structure to expose more binding tryptophan residues, resulting in lower peaks (Yi 2024).

#### 3.1.3 UV Spectra

Cur had a good UV absorption spectrum in the range of 420–450 nm, so the content of Cur in nanoparticles can be determined by measuring the UV spectrum of nanoparticles in this range. The UV spectra of the nanoparticles constructed with HMP/MMP/LMP were shown in Figure 2D–F. The figure showed that there were almost no UV absorption peaks when the concentrations of glutelin and pectin were 10:1 and RRCH4:1. As shown in Figure 1I, at these ratios, the Cur concentration in the supernatant was low because of the production of macroaggregates, thus resulting in UV spectra with a relatively low Cur content. At other ratios, the UV absorption peaks increased and then decreased with increasing pectin content. This finding indicated that the content of Cur in the nanoparticles also tended to increase but then decreases. The introduction of pectin increased the amount of negative charge and enhanced intermolecular repulsion; it changed the secondary structure of RGs, and when the pectin concentration reached a certain concentration, the interaction forces (e.g., electrostatic interactions, hydrogen bonding, and hydrophobic interactions) that constitute the nanoparticles reach equilibrium, which was optimal for encapsulating the active ingredient (Jiang et al. 2024). Above a certain concentration, the spatial structure formed was not conducive to the binding of the active ingredient to the active site of the protein, and the content of the encapsulated active ingredient decreased (Cheng et al. 2024). The optimal embedding ratios that emerged in this study were RRCH2:1, RRCM (4:1 ~ 1:1) and RRCL2:1.

Combining the above nanoparticle size, potential, FS, and UV spectroscopy results, we chose RRCH2:1, RRCM4:1, and RRCL2:1 as the optimal ratios for the next characterization, stability, and bioaccessibility studies.

### 3.2 EE and LC

The EE and LC of the nanoparticles were shown in Table 1, and the EE of glutelin encapsulated with Cur alone was (42.23 ± 0.28)%. After the addition of Rhas, the embedding efficiency significantly improved. Previous studies had also shown that the addition of Rhas can lead to the formation of a more compact protein structure, thereby significantly improving the EE. The nanocomposites formed after the combination of pectin, glutelin, and Rhas were used to encapsulate Cur, and the embedding efficiency of the ternary delivery system was approximately 72%, which was significantly lower than that of the binary delivery system constructed with glutelin and Rha solutions; this may be because the addition of negatively charged pectin changed the conformation of the protein, which was not conducive to the condensation of the protein, and more Cur could not be embedded to reduce the embedding efficiency, which was consistent with the results of Gao's study (Gao et al. 2024). It may also be because the concentration of the protein was lower than that of the control group, which did not provide more active ingredient binding sites.

**TABLE 1:** Encapsulation properties of RC/RRC/RRCH/RRCM/RRCL nanoparticles.

	EE	LC
RC	42.23 ± 0.28^c^	1.05 ± 0.0007^c^
RRC	89.60 ± 0.26^b^	1.53 ± 0.01^b^
RRCH	72.20 ± 0.36^a^	1.84 ± 0.009^a^
RRCM	71.70 ± 0.55^a^	1.83 ± 0.014^a^
RRCL	71.43 ± 0.47^a^	1.82 ± 0.012^a^

*Note:* The data are presented as mean ± standard deviation (*n* = 3), and significant differences (*p* < 0.05) are indicated by different letters.

Similarly, the addition of Rhas significantly increased the loading of Cur as compared to the addition of glutelin alone. However, the addition of pectin further increased the loading of Cur in the complexes, which was associated with a decrease in substrate concentration; according to the formulas for EE and LC, RRC had a higher EE due to a higher total amount of wall material, which allowed for a more efficient encapsulation of curcumin; however, the higher wall percentage also resulted in a decrease in the relative loading of curcumin per unit mass of the carrier, and thus a poorer LC performance. In contrast, RRCH, RRCM, and RRCL, due to the lower total amount of wall material, had higher LC despite a slightly lower EE, suggesting that they were able to load more curcumin with less carrier material. The loading of Cur in nanoparticles constructed from pectins with different degrees of esterification did not differ much, which was consistent with the results of encapsulation efficiency.

### 3.3 Driving Force for Nanoparticle Formation

Noncovalent interaction forces play an important role in the formation of nanoparticles. Hydrophobic forces drive protein folding and molecular recognition, hydrogen bonds stabilize secondary structures (α‐helices/β‐sheets), and electrostatic interactions direct tertiary organization. Disulfide bridges, while promoting aggregation, contribute to structural integrity. Denaturant‐specific turbidity assays (SDS: hydrophobic; urea: hydrogen bonds; DTT: disulfides) quantitatively revealed each interaction's relative contribution, where turbidity reduction magnitude was directly correlated with force dominance in nanoparticle stabilization. These findings establish a force hierarchy that dictates nanoscale architecture and functionality.

**FIGURE 3:**
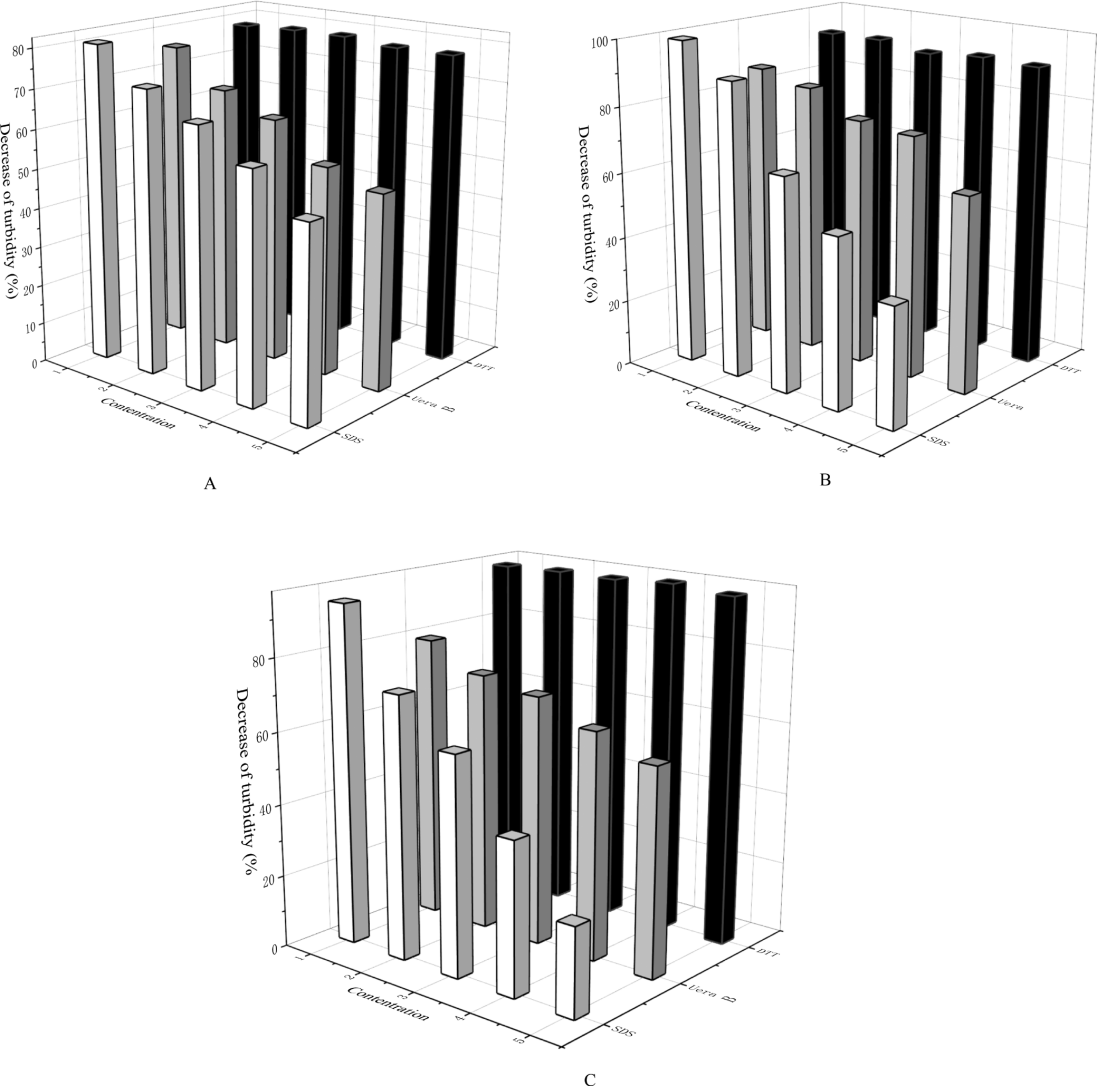
Effect of different denaturing reagents (SDS, Uera, and DTT) on the turbidity of RRCH (A)/RRCM (B)/RRCL (C) nanoparticles.

Figure 3A–C showed the trend of turbidity for RRCH2:1, RRCM4:1, and RRCL2:1 in the presence of different denaturants, respectively. The turbidity of the nanoparticles clearly decreased with increasing SDS content, indicating that hydrophobic interaction was involved in the formation of the nanoparticles. This effect gradually decreased with increasing pectin esterification, indicating that hydrophobic interaction was stronger in nanoparticles constructed from HMPs than in those constructed from LMP. The hydrophobic interactions of the nanoparticles constructed from HMP were stronger than those from LMP. Previous studies revealed that a high degree of methylation enhanced the hydrophobic interactions between protein‐pectin and protein–protein molecules in the sol–gel system, and this increased hydrophobic interaction may be related to the molecular weight and pKa of highly esterified pectin (He 2021; Wan 2019). The turbidity of the nanoparticle suspensions in the presence of urea followed the same trend as that in the presence of SDS, suggesting that hydrogen bonding played a key role in the formation of the nanoparticles. Specifically speaking, the decrease in turbidity with increasing urea content for different nanoparticles were RRCH (26.5% from 86.4% to 59.9%), RRCM (26.2% from 76.1% to 49.9%) and RRCL (21.6% from 79.2% to 57.6%). Thus, the more turbidity decreased with increasing urea content in RRCH relative to RRCL, namely, urea had a stronger destructive effect on RRCH; in other words, H‐bonding was weaker in RRCH than its effect in RRCL. This was related to the content of free hydroxyl groups in pectin, which was also proven by the study of Cheng (Cheng et al. 2024). However, the change in the concentration of DTT had no effect on the turbidity of the nanoparticles, suggesting that the formation of nanoparticles was not dependent on disulfide bonds. In summary, H‐bonding and hydrophobic interaction were involved in the construction of nanoparticles, where H‐bonding played a greater role in RRCL while hydrophobic interaction played a greater role in RRCH. It further illustrated the existence of a certain effect of pectin esterification degree on nanoparticle formation. Of course, changes in the polymer can also be characterized by changes in particle size in addition to changes in turbidity to determine the effect of different forces on nanoparticle formation (Li, Song, et al. 2024).

### 3.4 FTIR

**FIGURE 4:**
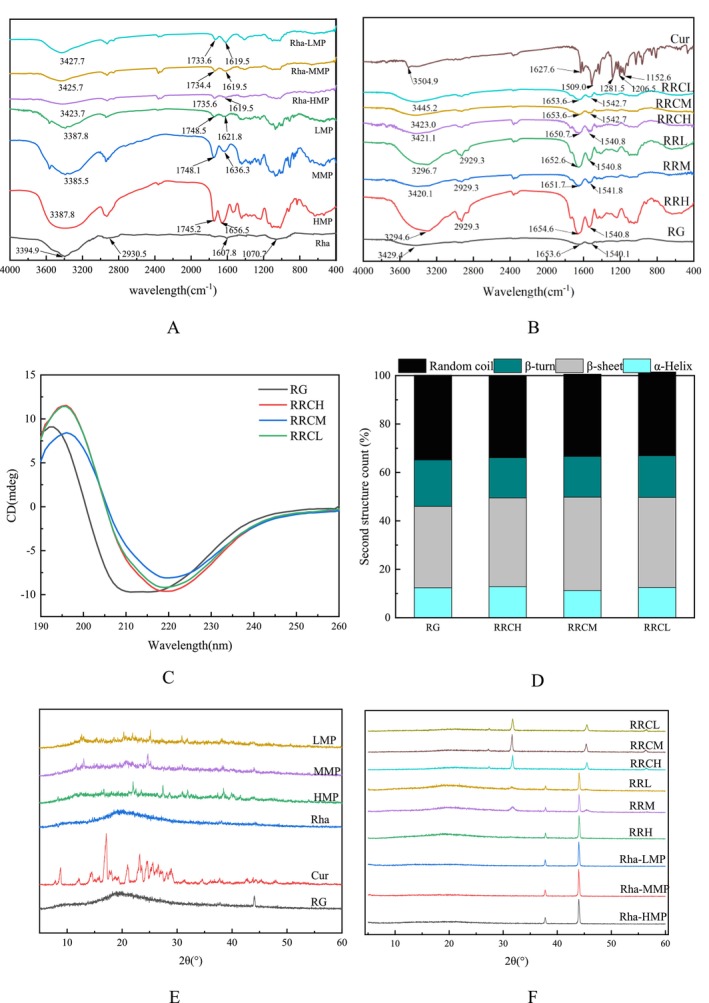
FTIR spectra (A, B). CD spectra (C) and changes in the secondary structure (D) and XRD patterns (E, F) of different nanoparticles.

The changes in functional groups were detected via FTIR spectroscopy to further analyze the intermolecular interaction forces, and the infrared spectra of different substances and nanoparticles are shown in Figure 4A,B. In the infrared spectrum of pectin (Figure 4A), the typical O‐H stretching peak appeared near 3387 cm^−1^; in addition, two other typical peaks were observed near 1748 and 1636 cm^−1^. The peak near 1748 cm^−1^ was attributed to the C=O stretching vibration, whereas the other peak was attributed to the undissociated carboxylic acid (COOH) stretching vibration (Guo et al. 2020). In the Rha spectrum, the peak at 3394.9 cm^−1^ was the O–H vibration, and the sharp peak at 2930.5 cm^−1^ was the vibration of the hydrocarbon (C–H) chain. The absorption peak at 1607 cm^−1^ indicated the presence of C=O in the ester compounds, and the absorption peak at 1070.7 cm^−1^ was also a characteristic peak of Rha (Dai, Li, et al. 2018). Upon the binding of Rhas to HMP/MMP/LMP, the telescopic vibrational fronts of ‐OH and C=O, as well as the vibration of undissociated ‐COOH, were displaced; this suggested that there was a combination of hydrogen bonding and hydrophobic and electrostatic interaction involved in the binding of the two.

Four typical peaks, 3429.4, 2925.4, 1653.6, and 1540.1 cm^−1^, were observed in the infrared spectra of RG (Figure 4B), which were related to the O‐H and N‐H stretching vibrations, the amide I band (C=O stretching vibration), and the amide II band (C‐N‐H stretching vibration), respectively, similar to the findings of Wu et al. (2024). After RG was bound to the above binary complexes, the O‐H vibrational peaks of RG shifted from 3429.4 to 3296.7, 3420.1, and 3294.7 cm^−1^, respectively, with increasing pectin esterification. The O‐H vibrational peaks of meso‐methoxylated pectin were less shifted, which could be attributed to the lower concentration of pectin as well as fewer free carboxyl groups. Moreover, the amide I band shifted from 1653.6 to 1652.6, 1651.7, and 1654.6 cm^−1^. Amide II, however, showed almost no displacement. This finding indicated that the binding of Rhas and pectin to RG occurs mainly through hydrogen bonding.

The characteristic peak of Cur appeared at 3504.9 cm^−1^, representing the O‐H stretching vibration of phenolic compounds, and at 1627.6 and 1509.0 cm^−1^, representing the C=O stretching vibration of allylic alcohol groups and the C‐C stretching vibration in aromatic rings, respectively. In addition, the peaks at 1428.0, 1281.5, and 1152.6 cm^−1^ correspond to the C‐H bending vibration, C‐O stretching vibration, and C‐O‐C stretching vibration of alcohols, respectively (Li, Wang, et al. 2024). Characteristic O‐H oscillatory shifts were observed upon complexation: RRL to RRCL (3296.7 → 3445.2 cm^−1^), RRM to RRCM (3420.1 → 3423.0 cm^−1^) and RRH to RRCH (3294.7 → 3421.1 cm^−1^), suggesting that Cur binds to the above ternary complexes via hydrogen bonding originating from the phenolic hydroxyl group of Cur with the hydrophilic head of Rhas as well as the N‐H group of proteins/carboxyl group of pectin molecules. The decreasing magnitude of shifts with increasing pectin esterification (RRCL > RRCH) suggested reduced hydrogen bonding in the highly esterified systems, which was consistent with their lower free hydroxyl availability. The lower O‐H vibrational shifts in RRCM may be related to the lower pectin concentration. Moreover, the peaks of amide I shifted from RRL to RRCL (1652.6 → 1653.6 cm^−1^), RRM to RRCM (1651.7 → 1653.6 cm^−1^) and RRH to RRCH (1654.6 → 1650.7 cm^−1^), and the peak of amide II shifted only slightly. These results suggested that hydrophobic and electrostatic interactions were also involved in the formation of nanoparticles but had fewer effects. The shift of amide I was attributed to the binding of the hydrophobic amino acids of glutamine to the hydrophobic tails of Rhas as well as to the hydrophobic sites of Cur through hydrophobic interaction. Overall, the hydrophobic interaction of the encapsulated Cur ternary delivery system gradually increased, and the electrostatic interactions gradually weakened with increasing pectin esterification; this was attributed to the number of free carboxyl groups in pectin. Gao verified that the presence of a surfactant (Rha) plays a synergistic role with polysaccharides in the stretching vibration of O‐H (Gao et al. 2024).

### 3.5 CD

Circular dichroism is usually used to characterize changes in the secondary structure of proteins, as shown in Figure 4C. The CD spectrum of RG had a maximum absorption front 191 to 193193 nm, indicating that there were many α‐helices in the secondary structure (Liang et al. 2021) and two negative peaks at 208 and 218 nm, reflecting randocoilsed and β‐sheet structures, respectively (Li, Wang, et al. 2024). A change in ellipticity indicated a change in the secondary structure of the protein. As shown in Figure 4D, with increasing pectin esterification, the content of α‐helices changed from 12.4% to 12.8%, 11.2%, and 12.5%, respectively. β‐folds increased from 33.6% to 36.7%, 38.6%, and 37.2%, respectively. β‐turn decreased from 19.2% to 16.6%, 16.8%, and 17.0%, respectively. The content of randcurlsurl was reduced from 35% to 33.9%, 34.0%, and 34.6%, respectively. The contents of α‐helices and β‐sheets in the nanoparticles constructed from pectin with different degrees of esterification increased, and the contents of β‐turns and irregular coils decreased, indicating that the addition of pectin promoted the gradual transformation of the RG structure from irregular to regular. The increased contents of α‐helices and β‐sheets in the nanocomposites also favored the formation of stable structures (Tabasi 2023). In addition, the content of β‐sheets increased more in the nanoparticles constructed by HMP, whereas the content of α‐helices increased more in the nanoparticles constructed by LMP, indicating that hydrophobic interactions were dominant in the nanoparticles constructed by HMP and that hydrogen bonds played a major role in the nanoparticles constructed by LMP because the formation of α‐helices and β‐sheets was related to hydrogen bonding and hydrophobic interaction. The hydrophilic carboxyl, hydroxyl, and amide groups of LMP can form hydrogen bonds with RG (Einhorn‐Stoll et al. 2021), and the hydrophobic methoxy groups in HMP can form hydrophobic interactions with hydrophobic amino acid residues in RG (Wang, Liu, et al. 2024). The circular dichroism results again demonstrated that hydrogen bonding and hydrophobic interactions were also two of the main drivers of nanoparticle composition.

### 3.6 XRD

X‐ray diffraction is often used to analyze the physical state of a substance to be measured. As shown in Figure 4F,G, the diffraction peaks of RG at approximately 10° and 20° are caused by the high α‐helix content of the protein polypeptide chain, confirming the amorphous nature of RG. Similarly, no very pronounced peaks were observed for pectins with different degrees of esterification, while Rhas only had a wide front at approximately 19°, which was attributed to their amorphous nature (Guo et al. 2020). The XRD profile of Cur showed many sharp and intense peaks in the range of 6°–30°, which was due to its highly crystalline structure. As shown in Figure 4G, after Rhas was combined with HMP/MMP/LMP, sharp peaks appeared only near 37.7° and 43.9°. When pectin and Rhas were combined, the smaller fronts near 37.7° may originate from the diffraction peaks of pectin here, whereas the sharp peaks near 43.9° may be due to the introduction of NaCl crystals during the preparation process. In addition, there are no other fronts, indicating that the binary complexes constructed from Rhas and pectins with different esterifications exist in an amorphous state. After the addition of RG, a characteristic peak of RG appeared near 20°, indicating that RG was successfully introduced. However, when Cur was encapsulated in the abovementioned ternary nanoparticles, the sharp peak corresponding to Cur disappeared. This finding indicated that Cur was successfully embedded in the nanoparticles and was present in the form of amorphous Cur. The diffraction peaks at approximately 32° and 45° may also be due to the introduction of NaCl during the formation of the nanoparticles.

Molecules with an amorphous morphology usually have a lower bond energy and, hence, have good solubility in water. At the same time, amorphous substances exhibit an exothermic reaction during the dissolution process, and more exothermic substances promote faster dissolution (Marabi et al. 2007). Therefore, the amorphous form of the nanoparticles contributes to their high solubility and sustainable release. Guo and Gao reported that the active ingredients in the crystalline form, such as Cur and lutein, were embedded in nanoparticles constructed with Rhas and polysaccharides, which became amorphous and had increased water solubility (Gao et al. 2024; Guo et al. 2020).

### 3.7 SEM

**FIGURE 5:**
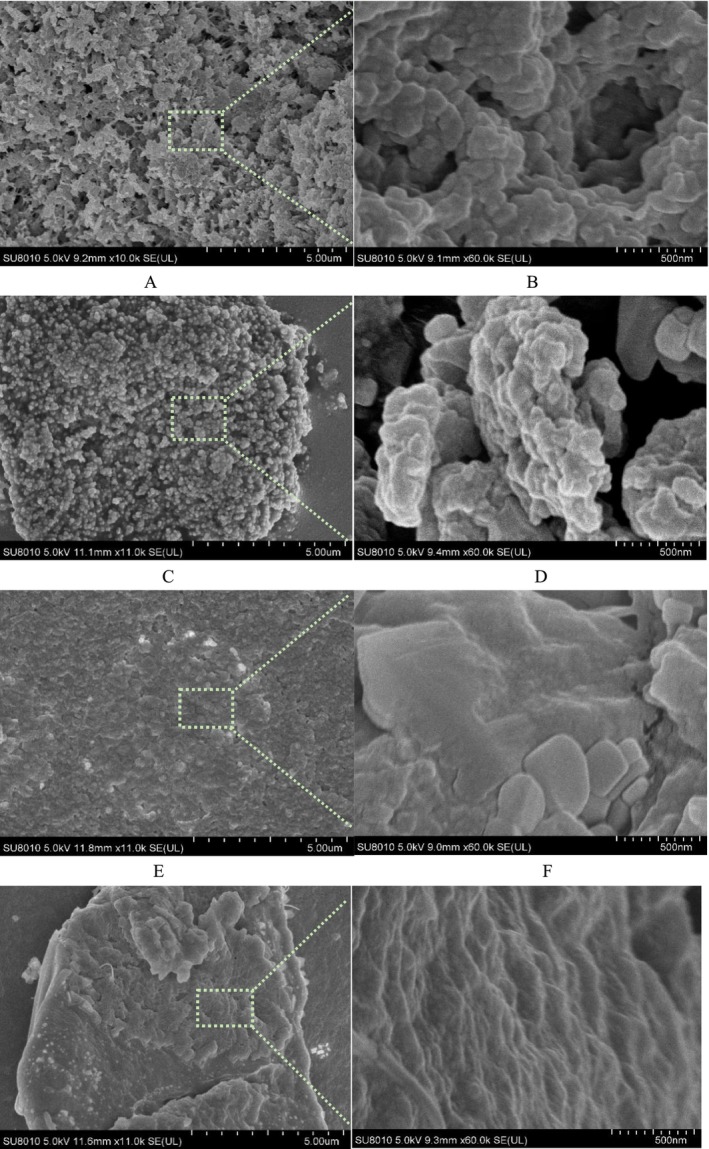
SEM images of RG (A, B) and RRCL (C, D)/RRCM (E, F)/RRCH (G, H) nanoparticles.

The microstructures of RG (A, B), RRCL (C, D), RRCM (E, F), and RRCH (G, H) were shown in Figure 5. SEM results revealed that the microstructure of RG was irregular, rough, and intertwined with a spherical structure, and the irregular particles were assembled into tight protein aggregates to form a rough and continuous spatial structure (Li et al. 2020). The cavity structure of RG facilitates the binding of active ingredients to it. Compared with the microstructure of RG, the Cur‐loaded nanoparticles had smaller particle sizes and a more uniform distribution. This can be explained by three factors. The addition of Rhas caused the proteins to form clusters, which made the nanoparticles more compact (Guo et al. 2021). However, the covalent and noncovalent interactions between proteins and polysaccharides could improve the functional properties of proteins and ensure their spatial stability (Zhang et al. 2021). Moreover, the introduction of hydrophobic active ingredients into the hydrophobic cavities of glutelin increased the steric hindrance of the nanoparticles (Geng et al. 2023). With increasing pectin esterification, the morphology of the nanoparticles gradually changed from distinct particles to cross‐links; this indicated that the degree of esterification of pectin affected the interaction between pectin and protein, which may be due to the lower negative charge on the surface of highly esterified pectin, which in turn resulted in lower electrostatic repulsion, increased the aggregation state, and affected its morphology. Moreover, the nanoparticles constructed with LMP had a dense and uniform structure due to their strong electrostatic attraction, and with increasing esterification, the particles in the system aggregated because HMP has high viscosity. Guo reported the same findings in a study of pea protein delivery systems constructed from pectin with different degrees of esterification; that is, with increasing pectin esterification, the aggregation of nanoparticles became increasingly obvious (Guo et al. 2020). Electron microscopy results revealed that the release of Cur from RRCH nanoparticles may be unfavorable due to the dense structure relative to that of RRCL/RRCM.

### 3.8 Photothermal and Storage Stability

Commercial products may be exposed to a series of complex environmental conditions (such as pH, light or temperature changes) during different processes of processing, storage, and transportation, among which light and heat are the main factors that promote Cur degradation. Therefore, studying the effects of these environmental pressures on the stability of nanoparticles is highly important.

#### 3.8.1 Light Stability

**FIGURE 6:**
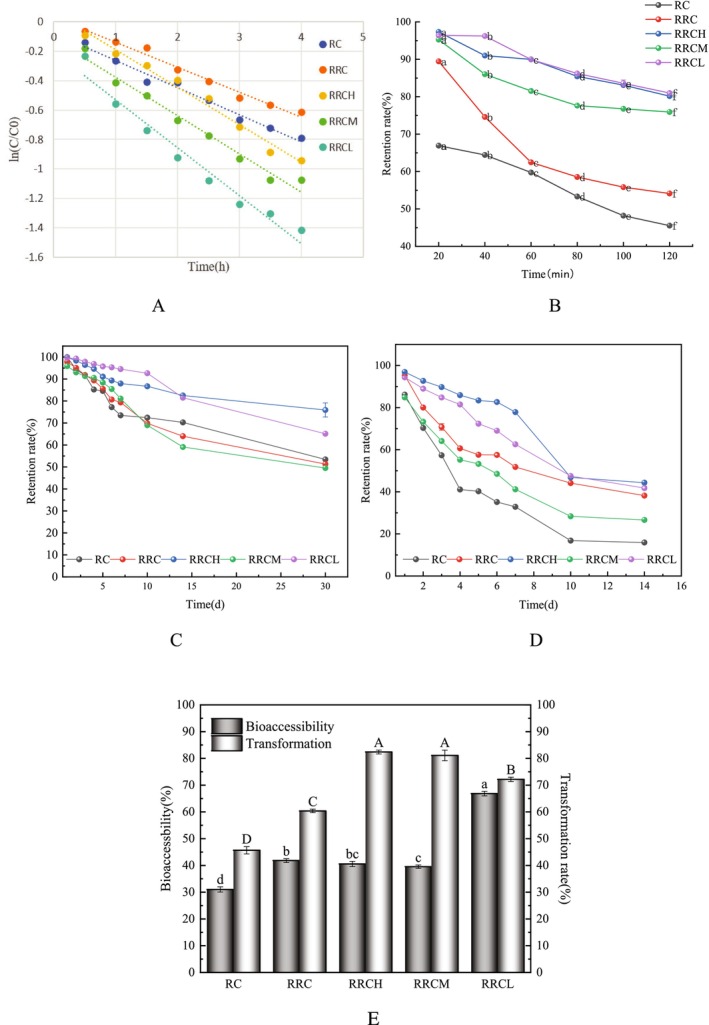
Photostability (A), thermal stabilization (B), and storage stability (C, D) of Cur in RC, RRC, RRCH, RRCM, and RRCL nanoparticles. Bioaccessibility of Cur in RC, RRC, RRCH, RRCM, and RRCL nanoparticles (E). The data are displayed as the means ± SDs (*n* = 3), with different letters denoting statistically significant differences (*p* < 0.05).

The photodegradation characteristics of the different nanoparticles embedded with Cur were shown in Figure 6A. When exposed to natural light, natural Cur degrades rapidly, forming smaller molecules such as vanillin, acetone, feruloylmethane, and ferulic acid (Liu et al. 2024). As shown in the figure, the *T*_1/2_ values of Cur in the RC/RRC/RRCH/RRCM/RRCL nanoparticles were 3.04, 4.75, 3.25, 2.14 and, 1.59 h (Table 2), respectively. The longer half‐life of the RC/RRC nanoparticles can be explained by two reasons: first, the rapid photodegradation of Cur was related to the destruction of the phototerene rings, and the higher content of RG in the RC/RRC nanoparticles during the construction process can provide more hydrophobic sites to bind to Cur. Second, the presence ofRhass altered the structure of hydrophobic domains in protein molecules, making it easier for aromatic rings to be inserted into protein molecules. Therefore, Cur can be better protected from natural light. The decrease in the half‐life of the nanoparticles after the addition of pectin occurred because the addition of pectin reduces the densification of the nanoparticles, which may be related to the decrease in the protein content during the preparation process. The half‐life of the RRCH/RRCM/RRCL nanoparticles gradually extended with increasing pectin esterification; specifically, the construction of nanoparticles with a high‐esterification degree of pectin was less likely to result in Cur degradation; this is because highly esterified pectin is more stable, which was consistent with Guo's findings (Guo et al. 2020).

**TABLE 2:** Degradation kinetic parameters of free RC, RRC, RRCH, RRCM, and RRCL nanoparticles.

Samples	*k* (h^−1^)	*t*_1/2_ (h)	*R* ^2^
RC	0.23157 ± 0.03226c	3.04 ± 0.40b	0.98
RRC	0.1483 ± 0.0201d	4.75 ± 0.65a	0.98
RRCH	0.2155 ± 0.02434c	3.25 ± 0.36b	0.98
RRCM	0.3292 ± 0.042966b	2.14 ± 0.26c	0.97
RRCL	0.4431 ± 0.065a	1.59 ± 0.23d	0.96

*Note:* The data are displayed as the means ± SDs (*n* = 3), with different letters denoting statistically significant differences (*p* < 0.05).

#### 3.8.2 Thermal Stability

In the food industry, heat treatment is often used to test the shelf stability of food products. Therefore, the effect of prolonged high‐temperature heating on the retention rate of Cur in nanoparticles was studied, as shown in Figure 6B. The retention of Cur gradually decreased under heat‐treated conditions, which was attributed to the fact that the degradation of Cur was closely related to its surrounding hydration layer, and heating promoted the interaction of the active groups of Cur with the aqueous phase, thereby accelerating the degradation of Cur (Liu et al. 2024). Moreover, heat disrupted the secondary structure of proteins, leading to the exposure of hydrophobic binding sites. The physical barrier formed by nanoparticles can better protect Cur from destruction. After heating for 120 min, the retention rate of Cur in RRC and RRC/RRCCM/RCL was still > 50%, especially after pectin was added. Pectin had a better protective effect on Cur, and the retention rate reached more than 70%, possibly because the viscosity of pectin can better stabilize the colloidal system. The protective effect of RRCH/RRCL nanoparticles on Cur was better than that of RRCM because the interaction force of Cur with RG, Rha, and pectin in RRCM was weaker than that of RRCH/RRCL nanoparticles, so it could not provide better protection for Cur.

#### 3.8.3 Storage Stability

Figure 6C,D depicted the variation in Cur retention of different nanoparticles embedded with Cur under refrigerated (4°C) and room temperature (approximately 30°C) conditions. As shown in Figure 6C, the retention rate of all the nanocomposites was greater than 50% after 30 days at 4°C, indicating that the nanoparticles had a good protective effect on Cur. Among them, the retention rate of Cur in RRCH was 75.92% ± 3.19%, followed by that of the RRCL nanocomposites, which was 65.10% ± 0.13%. Gao's study confirmed that the addition of Rhas and polysaccharides could improve the protective effect of nanoparticles on hydrophobic active ingredients (Gao et al. 2024). RRCH was more stable because of its greater adhesion and therefore provided better protection against Cur. Similarly, a similar trend was observed for the protective effect of the nanoparticles on Cur at room temperature (Figure 6D). These phenomena can be explained by the entry of Cur into the hydrophobic cavity of RG through hydrophobic interactions. The addition of Rhas could make the nanoparticles more compact. Pectin acted as a protective barrier to increase the stability of the nanoparticles. In conclusion, RRCH nanoparticles had a better protective effect on Cur compared with other embedded carriers. Also, the nanoparticles had better protection against curcumin at 4°C relative to storage at room temperature.

### 3.9 Bioaccessibility

During digestion, only the fat‐soluble active ingredient transferred to the bile salt micelles can be digested and absorbed in the human intestines, which means that the bioaccessibility of Cur depends on the amount of Cur retained in the micelles at the end of the final digestion. Previous studies had shown that free Cur had low bioaccessibility and conversion rates (Wang et al. 2022), which strongly limited its biological efficacy in the human body. In this study, Cur was embedded in nanoparticles, which greatly improved the bioaccessibility of Cur. As shown in Figure 6E, the bioaccessibility of Cur in RC/RRC/RRCH/RRCM/RRCL was (31.02 ± 0.97)%, (41.81 ± 0.73)%, (40.54 ± 0.96)%, (39.54 ± 0.62)% and (66.85 ± 0.85)%, respectively. The results showed that the bioavailability of Cur, especially the RRCL nanoparticles, was greatly improved after encapsulation. The improved bioavailability can be attributed to the following aspects. First, previous studies had shown that the presence of Rhas was beneficial for improving the stability of nanoparticles in the gastric phase (Chen et al. 2023). Second, Rhas has an amphiphilic structure, which effectively improved the solubilizing ability of the mixed micelles in small intestinal solution (Yuan et al. 2021). Finally, pectin acted as a protective barrier to bind to RG, which can effectively resist hydrolysis by pepsin and trypsin by shielding the hydrophobic portion of the center (Cheng et al. 2024). The greater bioaccessibility of the nanoparticles made from LMP than those made from HMP was attributed to the fact that the high viscosity of HMP would allow easy aggregation of the colloid, which would affect the digestion and absorption of Cur; this was in agreement with the results observed via SEM.

The transformation rate, which referred to the percentage of Cur that did not undergo biochemical or biological degradation after simulated digestion, was (45.6 ± 1.3)%, (60.36 ± 0.66)%, (82.39 ± 0.71)%, (81.11 ± 0.19)% and (72.17 ± 0.78)% for Cur in the nanoparticles, respectively. In contrast, pectin poly addition significantly enhanced the transformation of nanoparticles. Chang et al. reported that a zein‐based delivery system was more stable with the addition of pectin than without pectin under simulated gastrointestinal conditions (Chang et al. 2017); this was attributed to the fact that the formation of the ternary nanodelivery system relied on the combined participation of intermolecular hydrogen bonding, electrostatic interactions, and hydrophobic interactions. These interacting forces work together to maintain the stability of the nanoparticles, forming a barrier for better protection of Cur and delaying the degradation of Cur under simulated gastrointestinal conditions. Therefore, the nanodelivery system constructed in this study can serve as a better carrier for fat‐soluble active ingredients.

## 4 Conclusion

The FTIR, CD, and XRD results revealed that electrostatic, hydrogen bonding, and hydrophobic interactions were involved in the construction of the nanoparticles and that hydrogen bonding was stronger in RRCL, whereas hydrophobic interactions were stronger in RRCH. The SEM results revealed that the RRCL nanoparticles had better, more homogeneous external characteristics. Compared with the pectin‐free nanocomplexes, RRCH/RRCM/RRCL resulted in greater Cur conversion and better bioaccessibility of RRCL. The next step of research will focus on anti‐inflammatory and antioxidant studies as well as studies of the cytotoxicity of the nanoparticles. The RRCL delivery system constructed in this study has better bioaccessibility and can be used as a good carrier for hydrophobic active ingredients, which provides a new reference for its application in the biomedical and food industries.

## Author Contributions

**Lei Chen:** funding acquisition (equal), writing – review and editing (equal). **Qiongqiong Wu:** data curation (equal), methodology (equal), writing – original draft (equal). **Shaokang Li:** data curation (equal), formal analysis (equal). **Shuangli Wang:** data curation (equal). **Weidong Lin:** software (equal). **Xiaodan Zang:** funding acquisition (equal), project administration (equal), writing – review and editing (equal).

## Conflicts of Interest

The authors declare no conflicts of interest.

## Data Availability

The data supporting this study's findings are available from the corresponding author, XD Zang, upon reasonable request.
